# Serotype 1 pneumococcus: epidemiology, genomics, and disease mechanisms

**DOI:** 10.1016/j.tim.2021.11.007

**Published:** 2021-12-20

**Authors:** Chrispin Chaguza, Marie Yang, Laura C. Jacques, Stephen D. Bentley, Aras Kadioglu

**Affiliations:** 1 Parasites and Microbes Programme, Wellcome Sanger Institute, Wellcome Genome Campus, Hinxton, Cambridge, United Kingdom; 2 Darwin College, University of Cambridge, Silver Street, Cambridge, United Kingdom; 3 Department of Clinical Infection, Microbiology and Immunology, University of Liverpool, The Ronald Ross Building, West Derby St, Liverpool, United Kingdom; 4 NIHR Mucosal Pathogens Research Unit, Division of Infection and Immunity, University College London, London, United Kingdom; 5 Department of Pathology, University of Cambridge, Tennis Court Road, Cambridge, United Kingdom

**Keywords:** Pneumococcus, serotype 1, genomics, outbreaks, invasiveness, virulence

## Abstract

*Streptococcus pneumoniae* is a significant cause of morbidity and mortality worldwide, causing life-threatening diseases such as pneumonia, bacteraemia, and meningitis, with an annual death burden of over one million. Discovered over a century ago, pneumococcal serotype 1 (S1) is a significant cause of these life-threatening diseases. Our understanding of the epidemiology and biology of pneumococcal S1 has significantly improved over the past two decades, informing the development of preventative and surveillance strategies. However, many questions remain unanswered. Here, we review the current state of knowledge of pneumococcal S1, with a special emphasis on clinical epidemiology, genomics, and disease mechanisms.

## Introduction

*Streptococcus pneumoniae*, also known as the pneumococcus, is an α-haemolytic Grampositive, opportunistic pathogen which colonises the human nasopharynx [1]. Colonisation with the pneumococcus is typically asymptomatic and more common in infants compared to adults. Rates of colonisation tend to vary by age, for example, on average 7% and 11% prevalence in adults in the United Kingdom and Malawi, respectively, and approximately 42% in children [2,3] reaching rates as high as 100% during the first year of life [4]. Colonisation is also believed to be a pre-requisite for the onset of pneumococcal disease and may confer, albeit weak, natural immunity against pneumococcal infection [5,6]. The pneumococcus can spread from the nasopharynx to other sites of the body such as the lung, blood, and meninges, to cause both non-invasive and invasive pneumococcal diseases (IPD) [1]. Recent global estimates report that *S. pneumoniae* is the largest cause of death due to lower respiratory tract infections, resulting in over one million casualties annually [7,8], of which ca. 320,000 are children under the age of 5, affecting particularly low- and middle-income countries (LMICs) [8].

Thus far, 100 pneumococcal capsular serotypes have been identified [9,10]. Distinct capsular serotypes may differ in their ability to colonise the nasopharynx [11] or cause IPD [12]. Pneumococcal S1 was among the first serotypes discovered in the early 20^th^ century [13] and ranks among the most common serotypes associated with IPD globally - particularly in Sub-Saharan Africa (SSA), South America and Asia [14] - where it is widely associated with outbreaks (Table 1). Although bacteraemia, pneumonia, and meningitis are the main IPD caused by pneumococcal S1, it is also widely recovered in patients with empyema [15].

## Pneumococcal serotype 1 is an atypical commensal and an adept invader

The duration of pneumococcal carriage varies significantly across epidemiological settings - with age being one of the strongest determinants [11,16,17]. Unlike most pneumococcal serotypes, which are proficient asymptomatic colonisers presenting modest invasive potential, S1 typically exhibits high invasive disease potential i.e. invasiveness [18]. Invasiveness is classically defined as the odds ratio of IPD relative to asymptomatic nasopharyngeal carriage events, whereby a value >1 indicate a higher propensity of a given strain to be isolated in IPD patients vs. asymptomatic carriers. Several studies have consistently reported high invasive potential for pneumococcal S1 [18], temporally as well as geographically [19]. Studies have shown that S1 is more invasive compared to other serotypes, although the odds ratios vary globally. For example, - 9.6 in the United Kingdom [12], 15.6 in Belgium [20], 46.68 in Sweden [21], and 22.3 in Mozambique [22]. Such variable invasiveness estimates may reflect differences in sample sizes, host factors, circulating clones and distribution of serotypes within a given setting [14]. Despite high invasiveness - which translates to a high IPD burden - pneumococcal S1 may not necessarily be the most lethal strain e.g., compared to serotype 3 [23,24]. Current evidence on the lethality of pneumococcal serotypes primarily originates from high income settings where the incidence of S1 is low, hence further studies are warranted.

By virtue of its ability to establish nasopharyngeal colonisation over a very short duration (up to 1-2 weeks [11,16,25] compared to other serotypes, pneumococcal S1 does not appear to behave like a typical commensal [26,27]. Previous studies showed that the duration of carriage and invasive potential of pneumococci were positively correlated with the degree of encapsulation [28]. Indeed, the polysaccharide capsule is known to protect against immune-mediated clearance, e.g., by blocking the deposition and function of opsonins, evading neutrophil extracellular traps (NETs), or reducing mucus-mediated clearance. The zwitterionic structure of the pneumococcal S1 capsule is particularly intriguing in that surface charge-switching (i.e. zwitterionic) carriers are regarded as promising delivery systems to traverse the mucus layer and reach the underlying epithelium [29]. The S1 capsule was found to be significantly more resistant to opsonisation and complement deposition [30] with an enhanced capacity to translocate across the nasopharynx to reach the olfactory tissues and, ultimately, the brain compartments [31]. A rapid nose-to-brain translocation was reported to occur via an inward flow of fluid transporting *S. pneunoniae* through the cribriform plate and to the dura within minutes [32]. The hypervirulence of pneumococcal S1 was also found to be associated with the rapid release of pneumolysin and consequent enhanced dissemination [31,33]. Other serotypes, such as 5 and 7F, were also documented for their short duration of carriage and small capsule size [28]. Further investigations on the properties shared by all these serotypes may bring further insight into the peculiarity of S1.

## Pneumococcal serotype 1 outbreaks and epidemics

**Figure 1:**
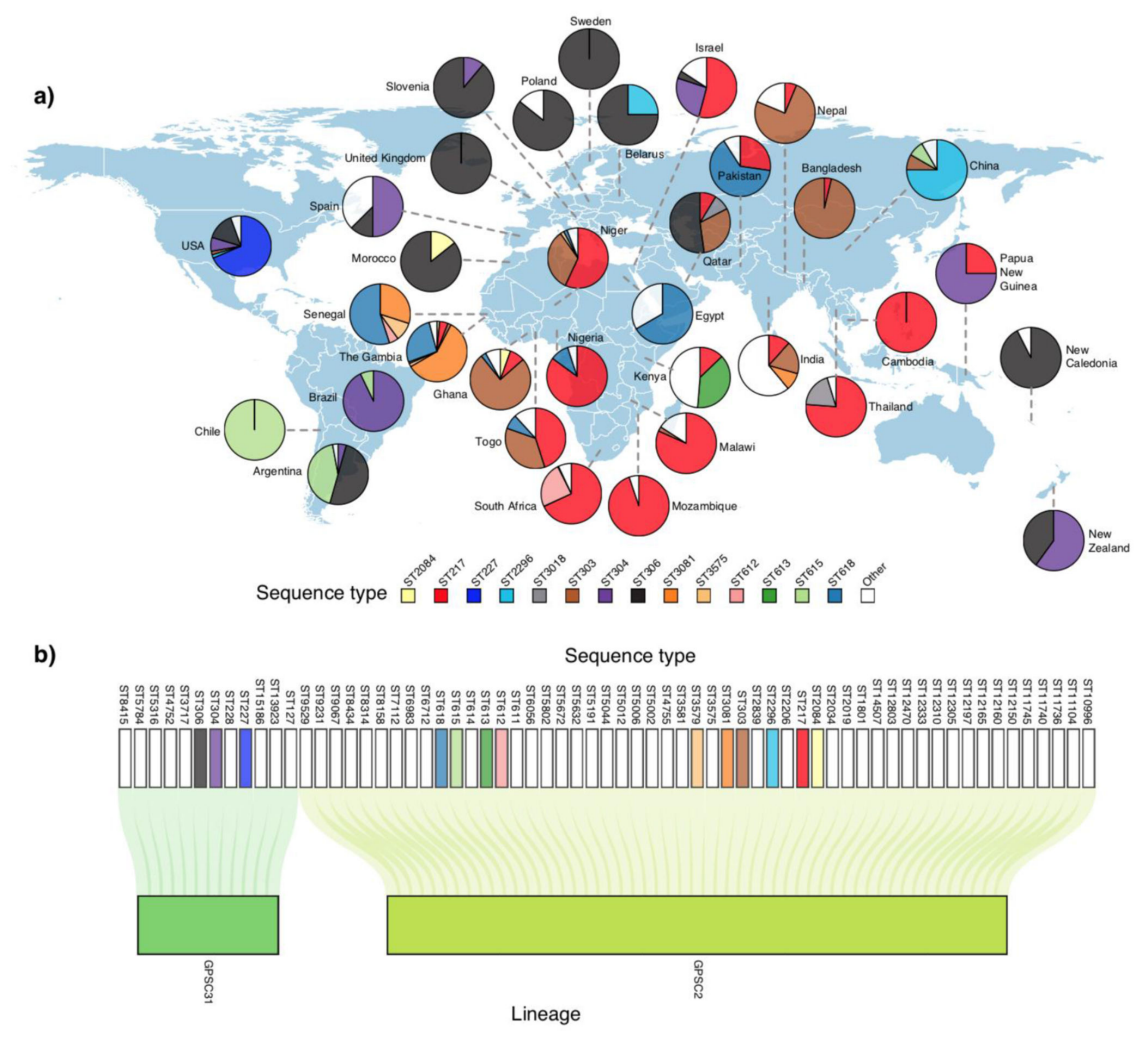
Serotype 1 sequence types (ST) and lineages globally. **a)** Geographical distribution of STs defined using MLST. The S1 isolates shown were obtained from both the Pneumococcal African Genomics (PAGe) [70] and Global Pneumococcal Sequencing (GPS) consortium projects [69]. The pie charts show the proportion of the STs in each country. The number of serotype 1 isolates used to estimate the proportion were small (less than 10) for some countries such as Slovenia, Spain, Poland, Morocco, Egypt, Peru, New Zealand, United Kingdom, Papua New Guinea, and Belarus. **b)** Association between serotype 1 STs and lineages defined using the GPSC nomenclature [69].

Although rare, outbreaks of pneumococcal disease do occur and are commonly associated with vulnerable groups, such as the homeless or alcoholic individuals [34], those living in closed communities or endemic regions such as the African meningitis belt. Although non-S1 serotypes have shown potential to cause similar outbreaks, a large majority of reported outbreaks were associated with S1 [35,36] and serotype 5 [37]. Interestingly, the two earliest recorded outbreaks of S1 were associated with lobar pneumonia and occurred at a boys asylum and at the Rochester State Hospital both in 1917 and in New York, USA [38] and resulted in fatality rates of 50% and 66%, respectively. Table 1 provides a detailed summary of known reported outbreaks of S1 globally. Despite the apparent increase in the number of outbreaks reported over the past 50 years - likely due to the increased availability of typing techniques and the expansion of global surveillance programmes, the widespread use of antibiotics and the rollout of pneumococcal vaccines have led to an overall decrease in the occurrence of outbreaks [39]. Based on current data, most of the reported pneumococcal S1 outbreaks seem to be associated with only a select number of sequence types (ST) (or clones) as defined by pneumococcal multilocus sequence typing or MLST (Table 1). Indeed, S1 outbreaks in Europe, North America, and South America are typically associated with ST306 clones, while in Africa, especially SSA, pneumococcal ST217 and its single locus variants - namely ST303 and ST612 - were documented as the main culprits. Worryingly, S1 outbreaks continue to occur in some parts of the world even after the introduction of PCVs, for example, in Ghana [40] and Central African Republic [41] in the West African meningitis belt. These outbreaks will likely become less common due to the expansion of routine immunisation programmes. Although the ST217 clone is predominantly found in both West and Southern Africa, it forms distinct clades, which suggest that this clone may present limited transmission and local adaptation [42] (Figure 1a). In-depth investigations of different outbreak-causing S1 strains using whole-genome sequencing (WGS) datasets have the potential to reveal genetic patterns associated with virulence.

## Global distribution of pneumococcal serotype 1 clones

To date, several clones associated with pneumococcal S1 have been characterised, and their distribution varies globally. These clones were primarily defined using molecular techniques such as MLST [60], enabling consistent definition of clones suitable for surveillance internationally. Whole genome sequencing (WGS) is rapidly becoming a more prevalent approach as it provides higher resolution and more in-depth genome-wide information [61]. Using the MLST approach, Brueggemann *et al*. showed that S1 isolates belong to geographically distinct sequence types (STs) which form three genetic clusters designated as lineage A, B, and C [62]; lineage A was exclusively found in North America and Europe, while lineage B was mainly identified in Africa and Israel, and lineage C in South America. Since then, further S1 lineage diversity was defined (Figure 1): ST306 lineage A expands across Europe, Australia, South Pacific, and North Africa; ST217 lineage Bis is found in SSA, Middle East, and Asia while ST303 lineage B in West Africa and Asia; ST304 lineage B in Oceania, Europe, and South America; and ST615 lineage C is mostly isolated in South America (Figure 1a). Other clones tend to be geographically restricted: for example, the ST613 clone is associated with Eastern Africa, while ST227 and ST2296 are commonly found in the U.S.A and China, respectively. Although the temporal distribution of the S1 clones is remarkably stable, replacement of clones has been noted in some countries, including in The Gambia where ST3081 superseded ST618 [63], in the United Kingdom where ST306 succeeded ST227 [64], and in Brazil where ST304 became the dominant clone (no major clone identified prior to that) [65]. With the increasing availability of WGS data, there has been a shift towards defining pneumococcal S1 clones and lineages using genome-wide data. However, there have been challenges in assigning consistent and epidemiologically meaningful nomenclature for the lineages, making it difficult to compare findings over different studies. To address this, a consistent international nomenclature for the pneumococcal lineages known as Global Pneumococcal Sequence Clusters (GPSC), was proposed by Gladstone *et al*. [66] using the PopPUNK framework [67]. Using this GPSC nomenclature, the most common lineages of S1 were found to be GPSC2 and GPSC31 (Figure 1b).

## Population genomics of pneumococcal serotype 1

Genomic studies have revealed a phylogeographically structured population of S1 with infrequent inter-mixing of isolates from different countries, suggesting a rare spread of clones between settings [68,69]. Whether such limited dissemination of clones between countries is a consequence of the rare and short duration of carriage remains to be investigated. As a naturally competent bacterium, the pneumococcus reshuffles its genomic DNA through a process known as recombination [70] . For example, recombination of the capsule biosynthesis genes may alter the antigenicity of the expressed capsule, i.e. capsule switching [71]. An important consequence of capsule switching is vaccine escape, a phenomenon which occurs when strains presenting capsules targeted by PCVs switch to an antigenically distinct capsule not targeted by existing PCV formulations [72]. Recombination in S1 is considered to be rare compared to other serotypes which are capable of establishing longer durations of nasopharyngeal carriage [27,28] . A stable colonisation may indeed prolong exposure to co-colonising pneumococcal strains or related species, hence increasing the likelihood of genetic exchange (Box 1) [28]. Experimental studies have reported challenges in transforming S1 strains using a suicide plasmid instead of linear DNA [73,74], possibly supporting the notion that recombination rates are low in these strains.

Phylogenetically, S1 isolates belong to genetically related lineages sharing a common ancestor. To date, no isolates bearing the S1 capsule have been found in other distinct lineages [66,69], further highlighting both the purported low recombination levels of pneumococcal S1, and the possibility that capsule biosynthesis loci from S1 strains are less likely to be taken up by other lineages to generate capsule-switched S1 lineages. One recent study by Lessa *et al*. has, nevertheless, found homologs of S1 specific capsule biosynthesis genes in *Streptococcus mitis*, a closely related commensal of the pneumococcus sharing overlapping niches [74]. This raises important questions regarding the contribution of commensal streptococci to natural immunity against S1 and other pneumococcal serotypes.

Acquired antimicrobial resistance (AMR) has been widely regarded as rare in S1 isolates and this was attributed to low recombination rates. Low AMR rates were indeed reported in S1 isolates originating from high-income countries (HICs); however, in other parts of the world, such as in SSA, higher AMR rates were documented. Multidrug resistance rates (MDR) among S1 isolates in Malawi was nearly 82%, the highest recorded for any serotype [75], in contrast with an MDR of 4% in South Africa [61]. Similarly, high AMR rates were reported among S1 for cotrimoxazole and tetracycline but not chloramphenicol, penicillin, and cefotaxime, which are widely used to treat pneumococcal diseases in The Gambia [54]. Further studies are required to understand the factors driving the differences in the AMR rates of S1.

Genome-wide association studies (GWAS) have paved the way for exploratory investigations to identify genomic variation likely to affect bacterial phenotypes, including disease susceptibility [76,77] and antimicrobial resistance [78]. For example, a recent study comparing S1 isolates collected from the cerebrospinal fluid vs. non-CNS tissues of IPD patients revealed statistically significant allelic variants within the gene encoding the surface-exposed choline-binding protein A (CbpA or PspC) associated with neurotropism [79]. Cornick *et al*. also used whole-genome analysis to investigate the species-wide distribution of vaccine candidate genes in a global collection of S1 isolates to inform vaccine design [77]. Other studies have combined genomic analysis and *in vivo* modelling to investigate phenotypic differences leading to the clonal replacement of ST618 with ST3081 S1 isolates in The Gambia [80].

## Dynamics of pneumococcal S1 colonisation, shedding and transmission

While acquisition rate and carriage duration are known to be serotype-dependent, an additional layer of complexity resides in the observation that the human nasopharynx can harbor multiple pneumococcal serotypes simultaneously. While multiple pneumococcal serotypes can either simultaneously or sequentially colonise the human nasopharynx [81], previous studies showed that the current colonising serotype usually prevails [82,83]. Population-based studies conducted on samples collected in Gambian infants reported that co-colonisation with multiple pneumococcal serotypes was observed in over 40% of infants at any given sampling time point [16]. S1 was found at a prevalence of 0.93% (compared to 11.42% for type 19A at the highest end) and was also frequently associated with several serotypes.

Using murine models, the propensity of S1 to colonise the nasopharynx was shown to be reduced in the presence of a prior colonizer such as serotype 19F or 6B [33]. In high transmission settings, the persistence of invasive disease and ongoing outbreaks caused by pneumococci S1 has raised questions over the need to introduce a booster dose [52,84]. It is generally accepted that pneumococcal transmission occurs primarily through indirect contact via inhalation of airborne droplets, mainly prevailing in high-density living settings, e.g., daycare centres, prisons, and nursing homes [85], and in the presence of concomitant viral respiratory tract infections [90]. The type and amount of capsular polysaccharide were shown to play a critical role in the dynamics of pneumococcal shedding and transmission [86]; however, other factors such as density and duration of colonisation, as well as *ex vivo* survival [87,88] are also contributing influences. Murine models have been developed to aid the understanding of pneumococcal transmission and disease susceptibility [82,89]. These animal models were primarily developed using lab-adapted strains such as serotype 2 (D39) and 4 (TIGR4), or clinical isolates such as serotypes 6A, 19F, 23F, 7F, and 14. An adult murine model of pneumococcal transmission has been developed [32] which could further the understanding of factors promoting transmission of S1 and other serotypes to aid in development of better control measures.

## High attack rate, pneumolysin and haemolytic activity

**Figure 2:**
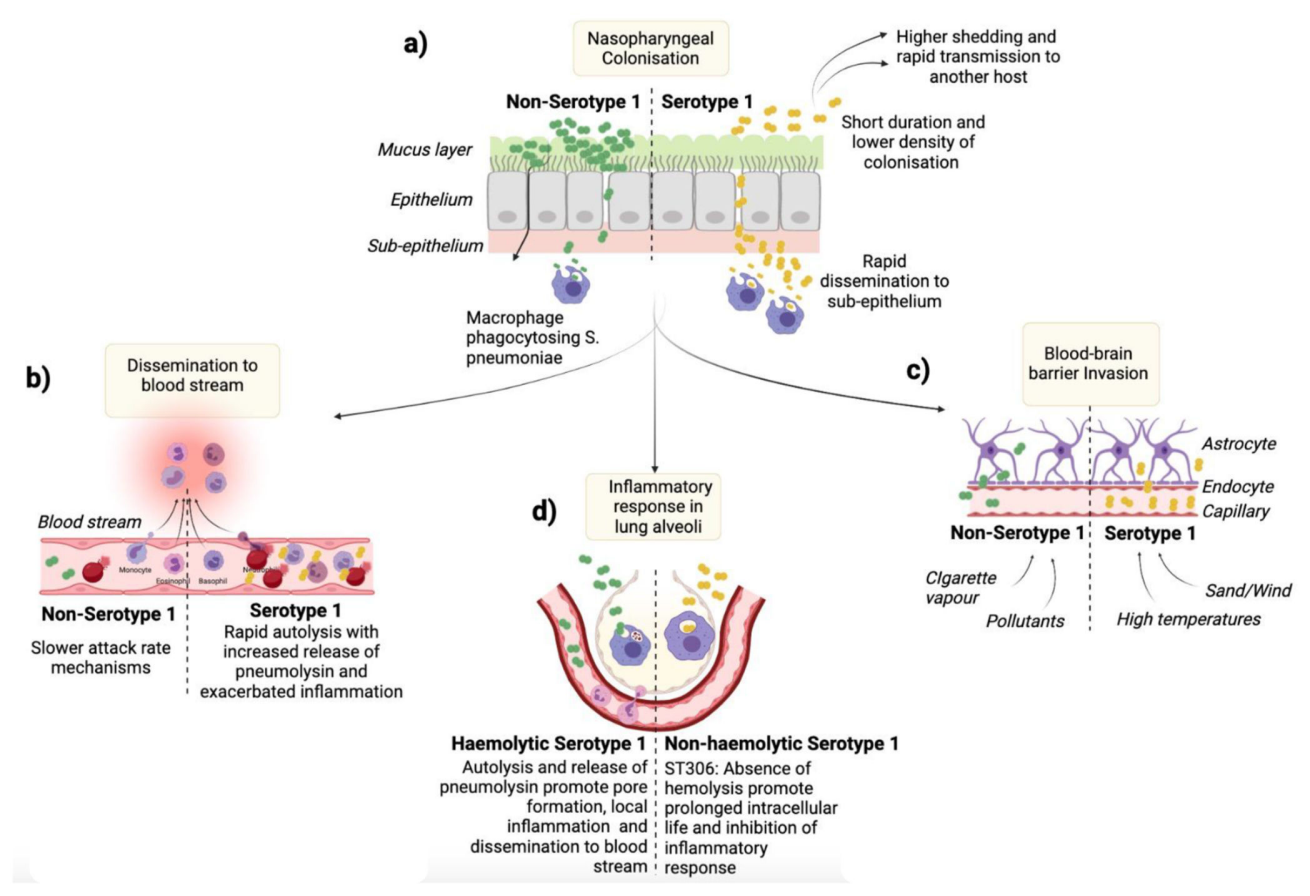
Hypothetical virulence mechanisms and host immunity to pneumococcal serotype 1. S1 is an atypical commensal known for its short duration (1-2 weeks) and lower density of colonisation in the human nasopharynx, potentially accompanied by higher shedding and host-to-host transmission (a). Its zwitterionic capsule promotes its ability to traverse the mucus layer and migrate deeper into the sub-epithelium to either reach the bloodstream - where it induces acute haemolysis due to its rapid autolysis (b), or cross the olfactory system and the blood-brain barrier to invade the central nervous system – especially in the presence of environmental factors such as sand, dust, and high temperature (c). The immune recognition of S1 and release of pneumolysin leads to the activation of inflammatory response that promotes the invasion process (d).

As early as 1937, Heffon found that S1 was responsible for 22% of pneumococcal pneumonia cases in children and 33% in adults, which is higher than the median attack rates of 7% seen in outbreaks of other pneumococcal serotypes [92]. The high attack rates of S1 could also be related to circumstances that may contribute to the development of IPD, such as high population density [93], viral co-infections [94] and environmental factors such as pollutants [95], cigarette vapour [96], airborne dust and high temperatures, as shown in Figure 2 [42]. In areas such as the African meningitis belt, the incidence of meningitis is over ten times higher than in Western Europe and the United States, with S1 accounting for 76% of all isolates causing meningitis [47]. Exposure of S1-colonised mice to high temperatures, representative of those in SSA, resulted in greater levels of bacterial dissemination and increased invasiveness [42]. Evidence suggests that the role of pneumolysin in S1 pathogenesis is complex, and mechanistic studies have been hampered by the inability to genetically modify S1. Recently, Terra *et al.* have successfully transformed S1 (ST5316, European lineage A) to deplete the pneumolysin gene [73]. Murine models of infection using this pneumolysin-deficient S1 suggest that S1 can still cause pneumonia in mice. Another isolate from lineage A (ST228), despite being cultured from the blood of a patient with pneumonia, was found not to express pneumolysin but could still disseminate from lungs into the blood, suggesting that other virulence factors play a more prominent role in the pathogenesis of these S1 sequence types. This contrasts with the depletion of pneumolysin in an ST615 clone which renders the isolate completely avirulent in murine models of pneumonia [97]. The pore-forming ability of pneumolysin was described as a critical virulence factor for pneumococci [98]. S1 strains expressing a fully haemolytic pneumolysin appear to induce higher secretion levels of type 1-interferon in mouse lungs, which drives dissemination into the blood, thus aiding pneumococcal S1 virulence [99]. In contrast, the absence of haemolytic activity was associated with reduced inflammation; for example, poor activation of the NLRP3 inflammasome, which drives IL-1β production and reduction in pro-inflammatory cytokines such as KC and IL-6, led to a decrease in neutrophil recruitment in the lungs [100]. Interestingly, however, S1 clones such as ST306 cause high rates of pneumococcal disease while expressing a non-cytolytic pneumolysin variant [64] and was commonly associated with a high incidence of non-lethal empyema, particularly in recurrent paediatric infections [101]. While the ST306 clone was first described almost two decades ago, the evolutionary significance of non-cytolytic pneumolysin variants remain elusive.

Badgujar *et al*. suggested that the loss of haemolytic activity enables ST306 to adopt an atypical intracellular lifestyle due to improved cellular invasion and attenuation of inflammatory responses, including autophagy evasion thus promoting long term survival in the lower airways [102]. Further work is warranted to clarify the contributory role of pneumolysin in S1 pathogenicity.

## Concluding remarks and future perspectives

Remarkable advances have been made regarding understanding the epidemiology and biology of pneumococcal S1, including the critical role of the antigenic outer cell wall polysaccharide capsule and virulence factors, such as pneumolysin and autolysin, on colonisation and disease. Nevertheless, challenges remain to fully understand the atypical hyper-invasiveness of S1 pneumococci compared to other serotypes. Recent advances in transforming S1 isolates will pave the way for further studies to examine the role of the capsule and genetic background in the invasiveness of pneumococcal strains. And although the IPD burden due to S1 has significantly decreased globally owing to the introduction of higher-valency PCVs (PCV10 and PCV13), it remains as important as ever to understand the determinants and mechanisms of virulence and pathogenicity of highly invasive pneumococcal serotypes such as S1. Such knowledge will inform the implementation of improved public health interventions, and the development of broader-acting vaccines to prevent IPD caused by serotypes other than those included in the existing PCV formulation, including those poorly controlled by PCVs e.g. serotype 3 [108] and niche replacement serotypes e.g. serotype 12F [109].

The increasing availability of omics (genomic, transcriptomics, proteomics, metabolomics) datasets of pneumococcal isolates, from small [68,110,111] and large-scale projects such as the PAGe [70] and GPS consortiums [66], will provide unprecedented opportunities to understand the complex epidemiology and biology of S1. Additionally, the development of *in vitro* and *in vivo* translational models closely mimicking human physiology, combined with the ability to genetically manipulate the pneumococcal S1 genome, will promote further understanding of the hyper-invasiveness of pneumococcal S1. Outstanding questions pertaining to pathogen, host and environmental factors will be addressed through an integrated approach encompassing multi- and interdisciplinary approaches combining epidemiological, bioinformatical, experimental and clinical studies. Ultimately, addressing these pertinent questions will inform prevention and control strategies for pneumococcal diseases.

## Figures and Tables

**Table 1:** Summary of known outbreaks and epidemics associated with pneumococcal serotype 1.

Year of outbreaks	Place of outbreak	Continent	Specific location	Outbreak setting	Clinical manifestation	Age group	Conjugate vaccination period	Dominant clone	Reference
1917	USA	North America	Rochester	Boys’ asylum and hospital	Pneumonia	All ages	Pre-PCV	Unknown	[38]
February to June 1931	Norway	Europe	Bærum county	Orphanage	Croupous pneumonia	Boys (5 to 20 years)	Pre-PCV	Unknown	[43]
January to March 1937	USA	North America	Worcester, Massachusetts	Hospital setting	Lobar pneumonia	Adults	Pre-PCV	Unknown	[44]
Early 1978	USA	North America	Boston city	Homeless men’s shelter	Bacteraemia	Adults	Pre-PCV	Unknown	[45]
April 1988 to March 1989	France	Europe	Paris	Homeless men’s shelters	Acute pneumonia	Adults	Pre-PCV	Unknown	[34]
Winter of 1991	Australia	Australia	Alice Springs region	Mainly Aboriginal men	Bacteraemia pneumonia	Children and adults	Pre-PCV	Unknown	[39]
March 1997	Israel	Asia	City of Dimona, Southern Israel	Closed community	Lobar pneumonia and bacteraemia	Children and young adults	Pre-PCV	Unknown	[46]
1998 to 2003	Ghana	Africa	Kassena-Nankana District, northern Ghana	Community	Meningococcal- like meningitis	All ages	Pre-PCV	ST217	[47]
August 2000 to 2002	Canada	North America	Nunavick, northern Canada	Community	Severe pneumonia	Adults (20 to 64 years)	Pre-PCV	Unknown	[48][49]
2000 to 2007	South Pacific	Oceania	New Caledonia, Wallis and Futuna, and French Polynesia	Community	Bacteraemia and pneumonia	All ages	Pre- PCV	ST306	[50]
August 2001 to April 2002	Tunisia	Africa	Tunis	Jail and community	Acute lower respiratory tract infection	Adults	Pre-PCV13	Unknown	[51]
May 2002 to February 2005	Burkina Faso	Africa	District 15, 22, and Houndé	Community	Meningitis	Children and adults	Pre-PCV13	ST618	[52]
Between 2002 and 2004	Australia	Australia	Tiwi islands	Aboriginal population	Unknown	Children and adults	Pre-PCV13	Unknown	[53]
2004 to 2006	The Gambia	Africa	Nation-wide	Community	Several IPD	All ages	Pre-PCV	ST217	[54]
10 to 13 October 2006	United Kingdom	Europe	North Tyneside	Primary school	Lobar pneumonia	Children	Pre-PCV13	Unknown	[36]
1 June 2008 to 31 May 2010	Netherlands	Europe	Nation-wide	Community	Several IPD	Young female adults	Pre-PCV13	Unknown	[55]
2010 to 2013	Australia	Australia	Northern and central Australia	Mostly indigenous population	Several IPD	All ages (median age 15 years)	Pre- and post-CV13	ST306	[56]
October 2010 to 2012	Australia	Australia	Central Australia	Community	Several IPD	Older children	PCV10	ST306	[35]
2000-2004, and 2010	Kenya	Africa	Kilifi	Community	Bacteraemia and meningitis	Children and young adults	Pre-PCV	Unknown	[57]
2006-2007, 2010, and 2015	Malawi	Africa	Blantyre	Community	Bacteraemia and meningitis	All ages	Pre-PCV, and post-PCV13	ST217	[58]
December 2015 to April 2016	Ghana	Africa	Brong-Ahafo region	Community	Meningitis	Older children and adults	Post-PCV13	ST217	[40]
2016 to 2017	Ghana	Africa	Northern and upper west region	Community	Meningitis	All ages	Post-PCV13	Unknown	[59]
October 2016 to April 2017	Central African Republic	Africa	North-western region	Community	Meningitis	All ages	Post-PCV13	Unknown	[41]

## Highlights

Pneumococcal serotype 1 (S1) is a major cause of invasive pneumococcal disease (IPD) globally, especially in Africa, South America, and Asia

Pneumococcal S1 strains are frequently found in the tissues and fluids of IPD patients while they are rarely isolated from the nasopharynx of asymptomatic carriers, including in high pneumococcal carriage and disease settings

A select number of S1 clones are frequently associated with outbreaks and epidemics, especially in closed communities and highly vulnerable groups

Whole genome sequencing (WGS) has provided valuable insights on the clones, lineages, evolutionary dynamics, and phylogeographic structure of pneumococcal S1 globally

## Box 1 Hypothetical scenarios for the rarity of serotype 1 in the nasopharynx

The paradox of how pneumococcal S1 persists in the human population despite its rare nasopharyngeal carriage rate, including in IPD endemic settings, remains unresolved [63]. Several hypothetical scenarios have been put forward. Firstly, S1 isolates may inactivate capsule expression enabling transient colonisation of the nasopharynx as a non-encapsulated non-typeable pneumococcus [90]. Such inactivation of the capsule would in turn reduce detection of S1 by widely used tests such as latex agglutination and Quellung reaction. However, in this scenario, due to the capsule biosynthesis locus remaining in the genome, pneumococcal S1 would still be detectable using molecular tests such as polymerase chain reaction (PCR). Secondly, it has also been speculated that S1 strains may immediately acquire the capsule of another serotype upon colonisation. This would result in infrequent detection of S1 during carriage as the strains may be disguised as a non-S1 serotype when typing is done using microbiological or PCR tests. However, this scenario is unlikely as capsule-switching is a rare event, and to date, there has been no evidence of capsule-switching between pneumococcal strains expressing an S1 capsule. A third scenario is that pneumococcal S1 can colonise in an alternative compartment of the human body, where it can seed to the nasopharynx to facilitate person-to-person transmission. There is increasing evidence, using murine models, to suggest that pneumococcal S1 colonises niches such as olfactory tissues. If this phenomenon also occurs in humans, then oral-nasopharyngeal swabs would likely be unable to detect pneumococci in these niches and account for reports of low S1 carriage prevalence [31,91].

## Box 2 Impact of PCV on the incidence of pneumococcal serotype 1 IPD

Pneumococcal S1 is one of the serotypes included in the existing PCV10 and PCV13 vaccine formulations. Since the introduction of PCV vaccines into infant immunisation programmes in 2010, there has been a substantial global reduction in S1 outbreaks and IPD in both immunised children and unvaccinated older children and adults. Consistent with earlier data on PCV effectiveness in low disease burden settings [103], recent data describe a 95% reduction in disease incidence in settings presenting high S1 disease burden, six years following the introduction of PCV10 or PCV13 [104–106] . S1 epidemics persist in some settings despite high vaccine uptake, for example, an increase of S1 IPD was documented five years after the introduction of PCV13 in Malawi [58]. In the African Meningitis belt, whilst the overall incidence of pneumococcal meningitis has decreased in children under the age of five, S1 persisted as the main causative agent [40,59,107]. There have been speculations that the persistence of S1 post-vaccination may be attributed to the “3+0” immunisation schedule (no booster), which may be less effective compared to high income settings where a booster dose is given in the second year of life [84]. Another contributing factor may be that unvaccinated older siblings may be the reservoir for transmission to younger infants. The cyclical nature of S1 disease outbreaks has made it challenging to assess the true impact of PCV13 and will require a longer monitoring period with accurate documentation on vaccine uptake. Further studies are required to assess the impact of different PCV vaccine coverage and schedules on the incidence of S1 disease.

## Outstanding questions

Can genome-wide association studies comparing isolates sampled from asymptomatic healthy individuals vs. IPD patients reveal further clues about the hyper-invasiveness and pathogenicity of pneumococcal S1?

In the longer term, how will the introduction of PCVs impact pneumococcal S1 disease incidence in outbreak settings, and will it drive the emergence of vaccine-escape S1 variants?

Given its seemingly low prevalence and short duration in nasopharyngeal colonisation, how does pneumococcal S1 persist in the human population as one of the prevailing causes of IPD?

If the transformation rate of pneumococcal S1 differs from that of other serotypes, what are the underlying determinants of these differences?

Does inter-serotype competition play a major role in the disease and carriage dynamics of pneumococcal S1 strains?

How do variations in lineages and sequence types determine the differences observed in the mortality rate or disease severity observed among different pneumococcal S1 strains?

What are the host and environmental factors that influence the transmission and global distribution of pneumococcal S1 lineages?

## Glossary

**Attack rate**: Refers to the proportion of susceptible individuals who get infected during a specified period, for example, during an outbreak

**Capsule biosynthesis locus**: A cluster of genes delineated by the *dexB* and *aliA* genes in the pneumococcal genome, which are involved in the synthesis of the serotype-defining antigenic polysaccharide surrounding pneumococcal cells

**Global pneumococcal sequence clusters (GPSC)**: Consistent international nomenclature for pneumococcal lineages defined by the Global Pneumococcal Sequencing (GPS) project

**Multilocus sequence typing (MLST)**: A portable method for identifying bacterial clones based on the unique combination of alleles of the internal fragments of specific house-keeping genes (usually seven) for each species

**Pneumococcal conjugate vaccine (PCV)**: A pneumococcal vaccine containing purified capsular polysaccharides of a subset of serotypes conjugated to a carrier protein to induce a robust immune response

**Pneumolysin**: A potent cholesterol-dependent cytolysin and key pneumococcal virulence factor, which creates pores in cell membranes and depending on its concentration, can result in the lysis of host cells.

**Sequence type (ST)**: An ST refers to a unique combination of alleles for the internal fragments of housekeeping genes used to define the MLST scheme of a bacterial species
